# Erysipeloid Leishmaniasis: A Case Series

**DOI:** 10.7759/cureus.39026

**Published:** 2023-05-15

**Authors:** Tuğba R Ekmekci, Hüsna Güder

**Affiliations:** 1 Dermatology, Maltepe University Faculty of Medicine, İstanbul, TUR

**Keywords:** atipic form, skin infection, glucantime, cutaneous leishmaniasis, erysipelas

## Abstract

Cutaneous leishmaniasis can present in many different clinical forms. Diagnosis of atypical forms is often delayed. It is useful to keep in mind the diagnosis of cutaneous leishmaniasis, a mimicking disease, to reduce unnecessary treatment and patient morbidity. Erysipeloid leishmaniasis should be considered when presented as long-term erysipelas-like lesions that do not respond to antibiotics. We want to present our five patients with erysipeloid leishmaniasis, one of the atypical clinical forms.

## Introduction

The classic presentation of cutaneous leishmaniasis lesions is erythematous papules or noduloulcerative lesions. It usually settles on the face and extremities and leaves a scar while healing. However, many atypical forms, including paronychial, fissure, chancriform, erysipeloid, lupoid, scar leishmaniasis, lupus erythematosus-like, eczematous, verrucous, sporotrichoid, lupoid, papular, psoriasiform, mucocutaneous, and panniculitic variants, have been defined [1,2]. Cutaneous leishmaniasis usually causes no pain or itching in lesions that can be single or multiple [2]. Erysipelas is a bacterial skin infection characterized by warm and slightly painful erythema in the skin with significant systemic symptoms. The incidence of the erysipeloid form of cutaneous leishmania is reported between 0.05% and 3.2% [3]. Erysipeloid leishmania is described as erythematous, indurated, bacterial infection-like lesions [4].

## Case presentation

Case 1

**Figure 1:**
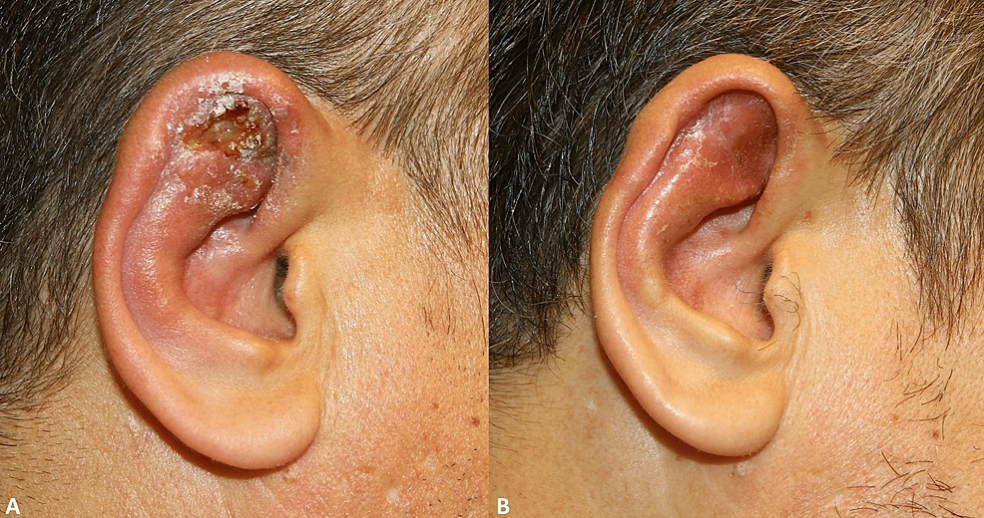
Before treatment (A) and 30th-day photo after treatment (B) of case 1.

A 46-year-old male patient with a complaint of crusting on his right ear for three weeks. Widespread redness, edema, tenderness, and ulceration with serous discharge in the upper outer part of the right auricle (Figure 1A). Leishmania bodies were seen in the smear examination. A skin biopsy showed dermal granulomatous infiltration and histiocytes containing Leishmania amastigotes. Leishmania spp. DNA-polymerase chain reaction (PCR) was positive. The patient, weighing 106 kg, was given 3 g/day of meglumine antimonate (Glucantime®) intramuscularly for 20 days and 900 mg/day of allopurinol for two months (Figure 1B).

Case 2

**Figure 2:**
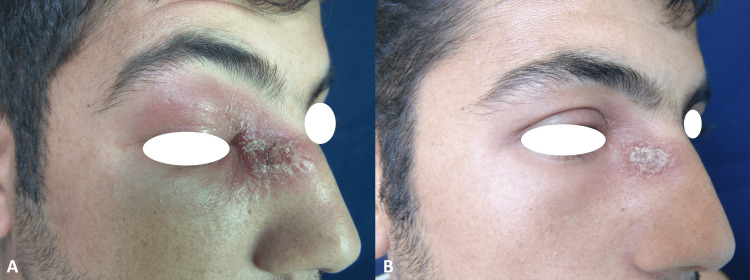
Before treatment (A) and 13th-day photo after treatment (B) of case 2.

A 26-year-old male patient who had a rash on his nose for two months. The patient had an erythematous infiltrated plaque 1.5-2 cm in diameter on the right side of the nose, along with redness and edema of the right eyelid with a few areas of yellow-brown crust (Figure 2A). The biopsy specimen showed epithelioid histiocytes and lymphocytes were forming granuloma structures. The diagnosis was confirmed by the demonstration of amastigotes in a slit smear. We observed significant improvement after 20 days of intramuscular treatment with meglumine antimonate 3 g/day (Figure 2B).

Case 3

**Figure 3:**
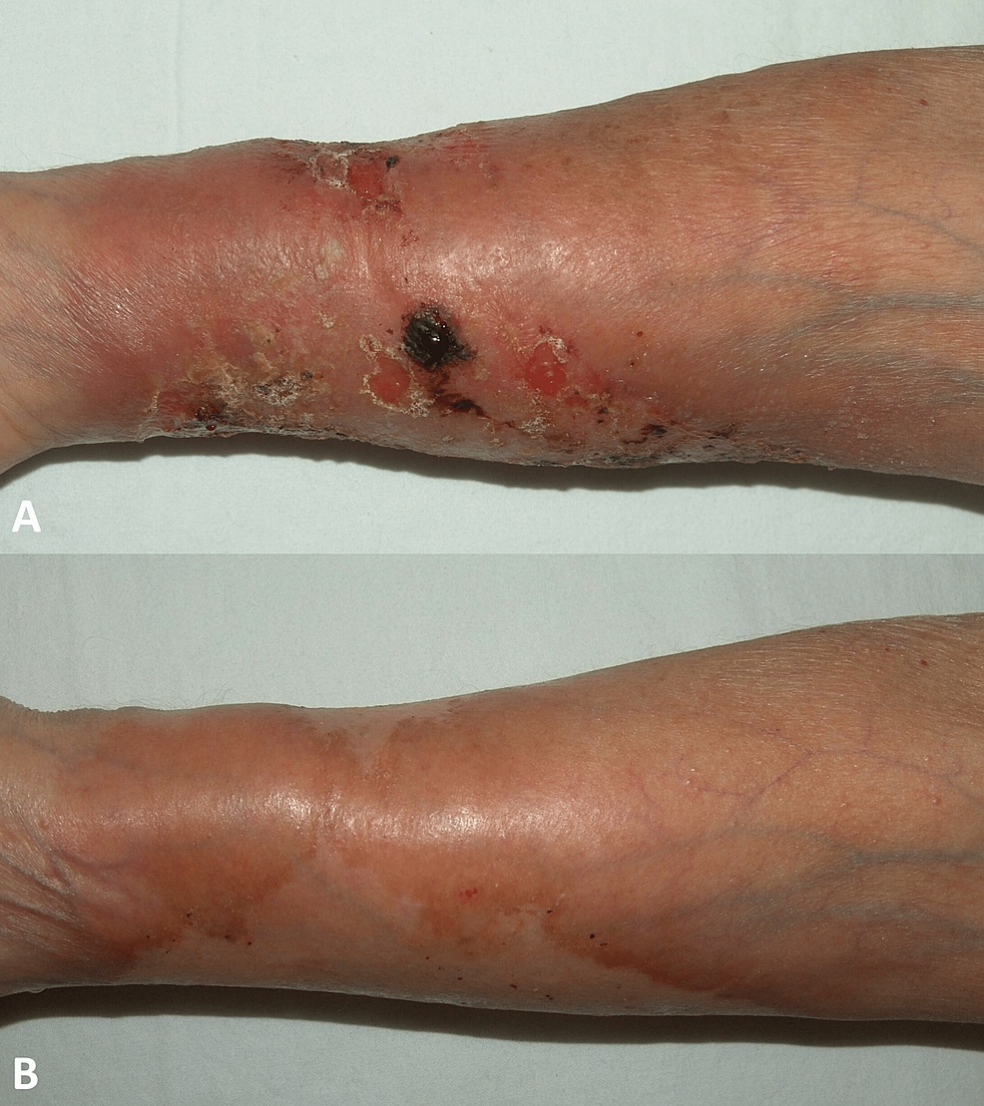
Before treatment (A) and 54th-day photo after treatment (B) of case 3.

A 76-year-old female patient who complained of a wound on her arm after being bitten by something five years ago. The wound continued to grow slowly, and a biopsy of the wound on his arm after one year was compatible with cutaneous leishmaniasis. The patient recovered after three sessions of intralesional and ten days of intramuscular meglumine antimonate treatment. She presented with the formation of swelling, redness, and crusting on the right arm for one month at the old lesion site. There was a circumferential erythematous, ulcerated lesion in the right arm's extensor surface (Figure 3A). Skin biopsy revealed a diffuse dermal infiltrate consisting of lymphocytes, histiocytes, plasma cells, and giant cells with granuloma formation. About 1.5 g/day of meglumine antimonate started. The treatment was given to the patient for 28 days. Systemic prednisolone 40-10 mg/day was given to the patient for ten days because of the lesion's erythema and itching. The patient also used allopurinol tablets, 3×300 mg (Figure 3B).

Case 4

**Figure 4:**
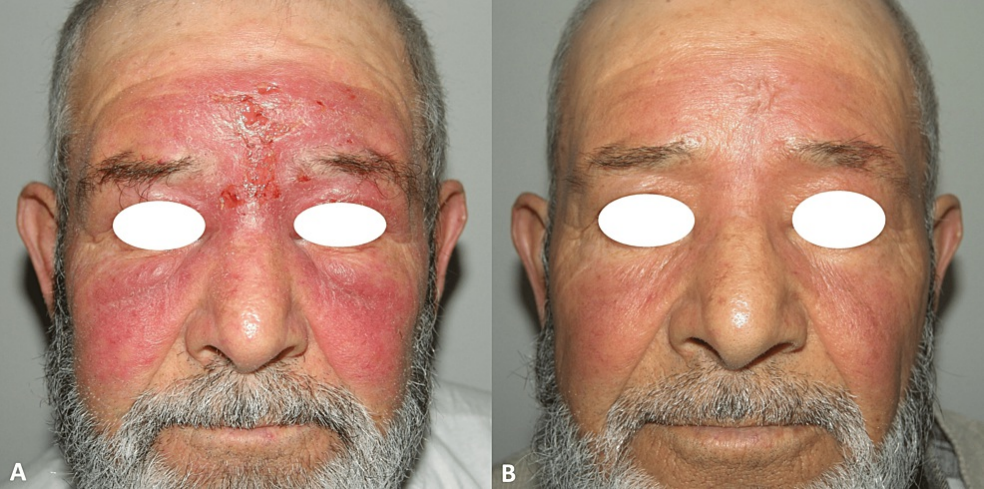
Before treatment (A) and 50th-day photo after treatment (B) of case 4.

A 72-year-old male patient who complained of a rash that started 14 months ago as a red nodule on his forehead and spread to the entire face. There was sharply demarcated erythematous scaling on the face, forehead, around the eyes, under the nasal root, and on the eyelids (Figure 4A). Dermal granulomatous infiltration with plasma cells was seen in the skin biopsy. The patient was treated with intramuscular meglumine antimonate 1.35 g/day for 20 days (Figure 4B). 

Case 5

**Figure 5:**
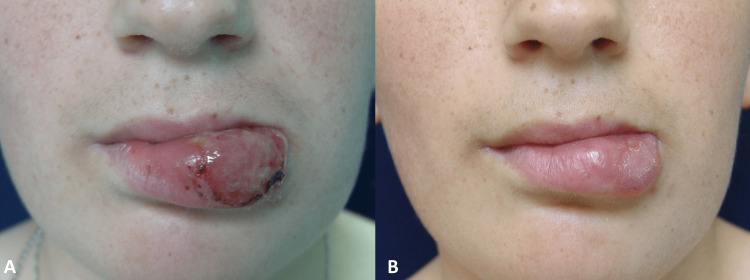
Before treatment (A) and 30th-day photo after treatment (B) of case 5.

A 16-year-old girl who had swelling and a wound on her lower lip for eight months. There was an erosive ulcerated lesion with prominent edema in the left half of the lower lip (Figure 5A). Poorly formed granulomas and diffuse dense dermal plasma cell infiltrate were found in the skin biopsy. The patient received meglumine antimonate 0.9 g/day intramuscularly and 600 mg/day of allopurinol for 30 days (Figure 5B). 

All patients received systemic antibiotic treatment several times with a preliminary diagnosis of bacterial infection, but no response was obtained. None of the patients had a fever or constitutional symptoms.

## Discussion

Leishmaniasis is endemic in many parts of Asia, Africa, America, and the Mediterranean. Erysipeloid leishmaniasis is generally reported in Iran, Pakistan, Turkey, and Tunisia. While cutaneous leishmania is seen as painless, dry papules and nodules, there are many atypical clinical forms. The erysipeloid type, one of these forms, is seen as an erythematous infiltrative plaque with long-term plaque lesions [4-7].

In an erysipeloid leishmania series of 40 patients, the largest series ever published, 55% of patients were over 55 years of age, and 62.5% were women [8]. It is claimed that erysipeloid leishmaniasis is more common in older women and that hormonal factors play a role [4,6]. As in our patients, the possibility of erysipeloid leishmania should be kept in mind in young and male patients.

Especially in atypical forms, diagnosis of the disease is difficult. Diagnostic methods are particularly important in this situation. Cutaneous leishmaniasis is diagnosed by observation of amastigotes in direct smears, culture, and histopathology. PCR and Montenegro's reaction are other diagnostic procedures [9].

The determination of amastigotes in the direct smear has been reported in the range of 30-96.23% [10]. The direct smear technique and the experience of the person obtaining are very important in seeing amastigotes. We did not see amastigotes in smears in cases 3, 4, and 5, where the disease duration was extended.

The sensitivity of a biopsy for diagnosis in Old World cases of cutaneous leishmaniasis is 59%. The sensitivity of seeing amastigotes is reported as 50% to 70%. It becomes more challenging to diagnose by biopsy in chronic cutaneous leishmaniasis lesions since there are fewer amastigotes. The most prominent features in cutaneous leishmania histopathology are non-necrotic histiocytic infiltration surrounded by lymphocytes and plasma cells, and mixed inflammatory infiltrates [11]. Adding leishmaniasis to the pre-diagnosis in biopsy examinations will make the pathologist's job easier.

It is crucial to take a travel history in the diagnosis of leishmaniasis. Cases 3 and 5 came to the city where they were diagnosed while living in an area endemic for leishmaniasis. Case 1 had visited the endemic area while living in the city where the diagnosis was made.

Although resistance is increasing in some regions, antimonial drugs (sodium stibogluconate and meglumine antimoniate) are still the first-line treatment option for most forms of leishmaniasis [11,12]. Among other systemic treatment options, amphotericin B, azoles, and miltefosine are among the most preferred [11].

The recommended treatment will be the meglumine antimonate intramuscular treatment option for prolonged, recurrent, resistance to therapy, and lesions located on the face, periarticular, and peri-orifice. Meglumine antimonate has proven its efficacy, but it is challenging to apply the treatment due to its side effects and not being readily available [9].

Systemic meglumine antimonate treatment was preferred in our patients for one or more reasons: facial location, periorificial location, prolonged, and recurrent disease. All of our patients received meglumine antimonate treatment intramuscularly for at least 20 days. Meglumine antimonate treatment was given by hospitalization in terms of follow-up of side effects. All patients achieved recovery without side effects and completed their treatment.

Meglumine antimonate intramuscular administration is difficult to apply in clinical practice for 20 days or longer. Since it is not enough to use a single vial when the patient is overweight, it is necessary to apply it to both gluteal areas on the same day. In the following days, significant sensitivity occurs in the gluteal region, making it difficult to continue the treatment.

Allopurinol, a xanthine oxidase inhibitor, showed a 74% improvement in cutaneous leishmaniasis in Asia. When allopurinol was added, it was shown to reduce the dose of antimony required for treatment [9]. We preferred to add allopurinol to the treatment to increase its efficacy without increasing the Glucantime® amount in our patients.

When cutaneous leishmaniasis treatment is complete, residual erythema may remain. Residual erythema does not mean that the treatment is insufficient; it disappears within months when patients are followed.

## Conclusions

Many factors may play a role in developing erysipeloid leishmaniasis, including parasite-related factors and factors related to the person's immune system. It seems complicated to reveal them due to factors such as the disease is not seen frequently outside of endemic areas and the identification of the parasite type is not easily accessible. In non-endemic areas, the diagnosis of leishmaniasis is often delayed. Erysipeloid leishmaniasis should be regarded as in long-term erysipelas-like lesions that do not respond to antibiotics. 

In cutaneous leishmania, diagnosis becomes difficult as the number of amastigotes decreases in long-standing lesions. Meglumine antimonate is still one of the best treatment options. Even after successful treatment, residual erythema may remain, which will subside within months.
